# Guiding adjuvant radiotherapy in stage III endometrial cancer: a prognostic model based on SEER

**DOI:** 10.3389/fonc.2024.1480102

**Published:** 2024-11-14

**Authors:** Chunmei Li, Zheshen Han, Linlin Chen, Gajincuo Du, Rong Cai

**Affiliations:** ^1^ Department of Radiation Oncology, Ruijin Hospital, Shanghai Jiaotong University School of Medicine, Shanghai, China; ^2^ Shanghai Key Laboratory of Proton-therapy, Shanghai, China; ^3^ School of Public Health, University of Hong Kong, Hong Kong, Hong Kong SAR, China; ^4^ Department of Radiation Therapy, Qinghai Provincial People’s Hospital, Qinghai, China

**Keywords:** nomogram, endometrial cancer, adjuvant radiotherapy, interaction, FIGO stage III

## Abstract

**Background:**

The effect of overall survival (OS) with adjuvant radiotherapy in stage III endometrial cancer (EC) remains controversial, and the adverse invents were unignorable.

**Methods:**

A total of 4,064 stage III EC patients who underwent adjuvant chemotherapy post-operatively were selected from Surveillance, Epidemiology, and End Results (SEER) Program. Independent risk factors were identified through Cox regression models. A nomogram was developed accordingly to predict OS. The concordance index (C-index), calibration, and Receiver Operating Characteristic (ROC) curves were applied to assess the model. Patients were divided into the low- and high-risk groups based on the optimal risk cutoff. Stratified analysis was conducted by radiation in both groups, and interactions between radiation and the risk groups were conducted to explore if any benefit less from adjuvant radiotherapy.

**Results:**

A total of five candidate factors were identified from the model showing good calibration and consistency discriminative power in the training (C-index: 0.73; 95% CI: 0.70–0.75), testing (C-index: 0.73; 95% CI: 0.69–0.77), and external validation cohorts (C-index: 0.88, 95% CI, 0.78–0.97). Patients were categorized into the low- and high-risk groups based on the optimal risk cutoff of 2.1048630. The women in the high-risk group experience significantly less (42% vs. 63% reduction) or none (0 vs. 63%) benefit (p-interaction = 0.049 vs. 0.016 in training and testing cohorts, respectively).

**Conclusion:**

A nomogram incorporating five variables was established to predict OS in stage III EC patients with adjuvant chemotherapy. The high-risk groups benefit less or none from adjuvant radiotherapy, which may serve as a useful reference for better guidance of radiotherapy in stage III EC patients.

## Introduction

Endometrial cancer (EC) is the most common gynecologic cancer in middle- or high-income countries, with an increasing incidence worldwide (1). Meanwhile, Federation International of Gynecology and Obstetrics (FIGO) staging III patients face a high risk of local recurrence and distant metastases, and the prognosis for recurrent patients is relatively poor, with a 5-year overall survival (OS) of about 57%–66% (2). Therefore, to reduce the recurrence rate and prolong survival, these patients should receive adjuvant therapy post-surgery. Based on several clinical trials (3–6), the revised 2023 guidelines on radiation therapy for EC by the American Society for Radiation Oncology (ASTRO) strongly recommended that all pathology types of stage III EC undergo postoperative adjuvant chemotherapy (7).

Several studies have demonstrated that external beam radiation therapy (EBRT) is effective in local control (LC) rates for stage III EC (5, 6, 8, 9). However, given the uncertain impact on OS, as demonstrated by PORTEC-3 showing a prolongation in OS with chemoradiotherapy post-operation while GOG 258 did not, the 2023 ASTRO guidelines suggest that adjuvant radiotherapy may be considered under specific conditions postoperatively with moderate-quality evidence (7). However, the condition of using adjuvant radiotherapy post-operation warrants further discussion. Similarly, the guidelines from the National Comprehensive Cancer Network (NCCN) have no clear definition with the recommendation that the postoperative adjuvant treatment approach for stage III EC being systemic therapy ± EBRT ± vaginal brachytherapy (10, 11). As for adverse events, EBRT was significantly linked to increased recurrences of urinary urgency, urinary incontinence, and bowel symptoms such as diarrhea and fecal leakage leading to limitations in daily activities as PORTEC-1 reported with a 15-year follow-up (12). Furthermore, the addition of concurrent chemotherapy exacerbated acute toxicity at 2 years, reporting a decline in functional levels and health-related quality of life (13), and at 5 years, 6% of patients still experience ≥Grade 2 sensory neuropathy toxicity persistently following chemoradiotherapy, characterized by significant tingling or numbness sensations (14).

Given the lack of a dependable model focusing on stage III EC patients for OS to test if any benefit less from adjuvant radiotherapy with the purpose of seeking opportunity for exemption from it, which could improve patients’ quality of life and reduce the medical burden, while still ensuring local-regional recurrence and OS, the study aims to construct a model as a reference to improve the guidance of adjuvant radiotherapy in stage III EC.

## Methods

### Study design and patients’ selection

**Figure 1:**
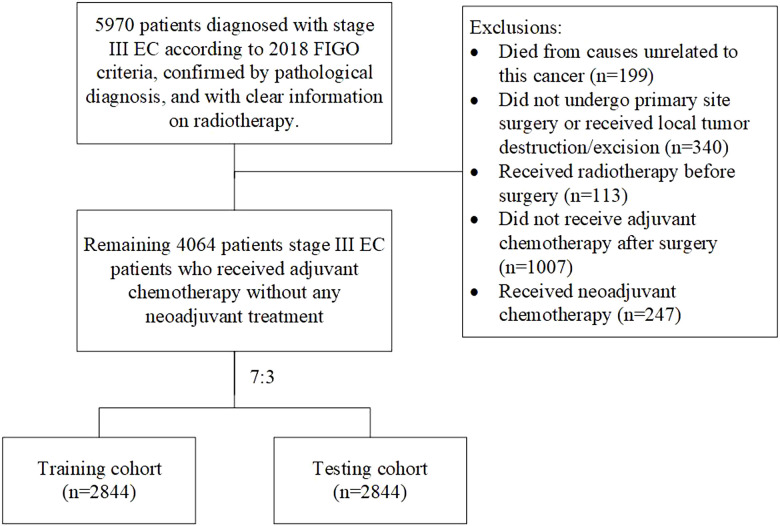
Study flow chart.

This retrospective cohort study identified stage III EC patients who underwent adjuvant chemotherapy and did not receive any neoadjuvant treatment before, diagnosed between 2018 and 2021 from Surveillance, Epidemiology, and End Results (SEER) Program (www.seer.cancer.gov) to seek patients who may not require adjuvant radiotherapy. Ethical review exemption was granted based on the anonymized nature of the data gathered from this public database. A total of 5,970 patients diagnosed with stage III EC according to the 2018 FIGO stage, confirmed by pathological diagnosis, and with clear information on radiotherapy were included in this study. Patients who died from causes unrelated to this cancer (*n* = 199), those who did not undergo primary site surgery or just received local tumor destruction/excision (*n* = 340), those who received radiotherapy before surgery (*n* = 113), those who did not receive adjuvant chemotherapy after surgery (*n* = 1,007), and those who received neoadjuvant chemotherapy (*n* = 247) were excluded from the study. **Figure 1** illustrates the inclusion and exclusion criteria. Ultimately, 4,064 patients met the inclusion and exclusion criteria were identified. Owing to that OS was the only survival outcome recorded in SEER and the previously established role of adjuvant radiation in LC, the primary outcome was OS. For all the datasets, if missing values were less than 15%, multivariate multiple imputation was conducted through the mice package in R software to enhance statistical power and mitigate potential biases that could arise from excluding women with missing data from the analysis.

### Study covariate and definition of cohort

The disease records of EC patients such as age, race, time from diagnosis to treatment, 2018 FIGO stage, grade, histology, tumor size, number of harvest lymph nodes (LN) in surgery, number of positive LN and pelvic LN, adjuvant radiation information, OS, and survival status had been accumulated in SEER data (**Supplementary Table S1**). According to the previous study (15) and the risk classification of histology, all EC patients were divided into two groups: (1) hormone-dependent endometrioid carcinoma with better prognosis, (2) non-hormone-dependent special pathological types with poorer prognosis along with rare pathological histological types combined under the label “Others.” Similarly, all EC patients were divided into two groups based on pathological classification: (1) stages I–II, (2) stage III, poorer differentiated and prognosis. Then, we randomly split all of the patients retrieved into training and testing cohorts at a 7:3 ratio using R software, following the methodology described in prior research (16, 17). Ultimately, the training cohort consisted of 2,844 individuals, while the testing cohort comprised 1,220 individuals.

### External validation

To assess the reliability of this model, 69 patients diagnosed with 2018 FIGO stage III EC between 2017 and 2022 in Ruijin hospital were recruited and evaluated independently. Patients’ information, including age, histology, grade, stage, number of positive LN and pelvic LN, and details of adjuvant radiation were gathered (**Table 1**). In the external validation, we similarly constructed a model with the same candidate variables as the training cohort. The accuracy of the model was evaluated using the concordance index (C-index), calibration curve, and ROC curve. Finally, we included and compared the related immunohistochemistry of EC, such as Ki67 percentage, P53 status, ER status, PR status, as well as MLH1, MSH2, MSH6, and PMS2, stratified by risk score group shown in **Table 1**.

**Table 1:** Demographic and clinico-pathological characteristics in the training, testing, and external validation cohorts.

	Level	Overall	Train	Test	*p*
*N*		4064	2844	1220	
Race (%)	White	3006 (74.0)	2106 (74.1)	900 (73.8)	0.952
	Black	565 (13.9)	396 (13.9)	169 (13.9)	
	Others	493 (12.1)	342 (12.0)	151 (12.4)	
Age [median (IQR)]		64.00 [57.00, 71.00]	64.00 [58.00, 71.00]	64.00 [57.00, 71.00]	0.308
Time_treatment [mean (SD)]		36.51 (37.76)	36.19 (36.62)	37.24 (40.29)	0.414
FIGO stage (%)	IIIA	981 (24.1)	693 (24.4)	288 (23.6)	0.302
	IIIB	326 (8.0)	217 (7.6)	109 (8.9)	
	IIIC1	2097 (51.6)	1484 (52.2)	613 (50.2)	
	IIIC2	660 (16.2)	450 (15.8)	210 (17.2)	
Histology (%)	Endometrioid carcinoma	2116 (52.1)	1485 (52.2)	631 (51.7)	0.799
	Others	1948 (47.9)	1359 (47.8)	589 (48.3)	
Grade (%)	I–II	1863 (45.8)	1303 (45.8)	560 (45.9)	0.987
	III	2201 (54.2)	1541 (54.2)	660 (54.1)	
Size [mean (SD)]		59.30 (37.51)	58.93 (32.82)	60.18 (46.66)	0.329
LN_Sur [median (IQR)]		2.00 [0.00, 2.00]	2.00 [0.00, 2.00]	2.00 [0.00, 2.00]	0.32
PosPLN [median (IQR)]		1.00 [0.00, 2.00]	1.00 [0.00, 2.00]	1.00 [0.00, 2.00]	0.247
PosLN [median (IQR)]		1.00 [0.00, 3.00]	1.00 [0.00, 3.00]	1.00 [1.00, 3.00]	0.659
RT (%)	None RT	1252 (30.8)	887 (31.2)	365 (29.9)	0.443
	RT	2812 (69.2)	1957 (68.8)	855 (70.1)	
RT_recode (%)	None RT	1252 (30.8)	887 (31.2)	365 (29.9)	0.758
	EBRT	1578 (38.8)	1107 (38.9)	471 (38.6)	
	VBT	414 (10.2)	285 (10.0)	129 (10.6)	
	Combined	820 (20.2)	565 (19.9)	255 (20.9)	
Our institution	Level	Overall	Low risk	High risk	*p*
*N*		69	53	16	
Age [median (IQR)]		60.00 [53.00, 65.00]	60.00 [52.00, 65.00]	58.00 [55.00, 66.00]	0.887
Histology (%)	Endometrioid carcinoma	44 (63.8)	44 (83.0)	0 (0.0)	< 0.001*
	Others	25 (36.2)	9 (17.0)	16 (100.0)	
Grade (%)	I–II	43 (62.3)	33 (62.3)	10 (62.5)	1
	III	26 (37.7)	20 (37.7)	6 (37.5)	
PosPLN [median (IQR)]		1.00 [0.00, 2.00]	1.00 [0.00, 2.00]	1.00 [0.00, 2.00]	0.663
PosLN [median (IQR)]		1.00 [1.00, 3.00]	1.00 [0.00, 3.00]	3.00 [1.00, 11.25]	0.107
FIGO stage (%)	IIIA	8 (11.6)	6 (11.3)	2 (12.5)	0.002
	IIIB	7 (10.1)	6 (11.3)	1 (6.2)	
	IIIC1	32 (46.4)	30 (56.6)	2 (12.5)	
	IIIC2	22 (31.9)	11 (20.8)	11 (68.8)	
RT (%)	None RT	15 (21.7)	8 (15.1)	7 (43.8)	0.037
	RT	54 (78.3)	45 (84.9)	9 (56.2)	
MLH1 (%)	No express	3 (11.5)	2 (14.3)	1 (8.3)	1*
	Express	23 (88.5)	12 (85.7)	11 (91.7)	
MSH2 (%)	No express	1 (3.8)	1 (7.1)	0 (0.0)	1*
	Express	25 (96.2)	13 (92.9)	12 (100.0)	
MSH6 (%)	No express	1 (3.8)	1 (7.1)	0 (0.0)	1*
	Express	25 (96.2)	13 (92.9)	12 (100.0)	
PMS2 (%)	No express	4 (15.4)	2 (14.3)	2 (16.7)	1*
	Express	22 (84.6)	12 (85.7)	10 (83.3)	
Ki67 percentage [mean (SD)]		54.81 (23.47)	55.71 (23.67)	52.38 (23.68)	0.667
ER status (%)	No express	12 (25.5)	7 (20.0)	5 (41.7)	0.248*
	Express	35 (74.5)	28 (80.0)	7 (58.3)	
PR status (%)	No express	18 (39.1)	10 (29.4)	8 (66.7)	0.038*
	Express	28 (60.9)	24 (70.6)	4 (33.3)	
P53 status (%)	normal	27 (60.0)	22 (66.7)	5 (41.7)	0.175*
	abnormal	18 (40.0)	11 (33.3)	7 (58.3)	

Time_treatment, time from diagnosis to treatment; FIGO, Federation International of Gynecology and Obstetrics. LN_sur, numbers of lymph nodes removed surgically. Pos_PLN, numbers of positive pelvic lymph nodes. posLN, numbers of positive lymph nodes. RT, radiation. *Fisher exact test.

### Statistical analysis

Categorical variables were expressed as percentages in comparison among the training and testing groups using either Pearson’s chi-squared test or Fisher’s exact test, while the t-test was used for continuous variables. Univariate and stepwise multivariate Cox regression models for OS were used to determine independent risk factors in the training cohort. In the univariate Cox model, if the *P* < 0.1, related factors would be retained for subsequent stepwise multivariate Cox regression to identify the ultimate candidate risk factors. Subsequently, we integrated these factors to construct a predictive nomogram to predict patients’ OS at 1, 2, and 3 years in the training cohort. The corresponding C-index, calibration curves, and ROC curves were used to measure the discriminatory ability of the model in the training and testing cohorts. Then patients were grouped into two risk classifications based on the optimal cutoff of the risk score derived from the independent risk factors in the nomogram. Kaplan–Meier curves and the log-rank test were used to conduct survival analysis to evaluate patients’ OS stratified by radiation in both risk groups. Subsequently, we carried out interactions between the risk score groups and radiotherapy with the method of Cox regression in both cohorts. All analysis was conducted using R (version 4.3), and a two-sided *P*- value < 0.05 was considered statistically significant.

## Results

### Characteristics of patients

Four thousand sixty-four individuals meeting criteria retrieved from SEER were divided into training (*N* = 2,844) and testing (*N* = 1,220) groups. The median follow-up duration for all patients was 18 months (interquartile range [IQR], 9–31 months). The median age of these patients was 64 years (IQR, 57–71 years), showing most of them were elderly women. Overall, 74% of the patients were Whites, and 12.1% were Asian or Pacific Islanders. The distribution of variables was balanced in the training cohort and testing cohort. In the training cohort, those with stage IIIC1 constituted a triple proportion than those with stage IIIC2 disease (52.2% vs. 15.8%). Moreover, patients diagnosed with histological endometrioid carcinoma (52.2%) and grade III (54.2%) collectively accounted for half of the training cohort. Moreover, the median number of positive LN was 1 (IQR, 0–1). Regarding treatment, approximately one-third of patients did not receive adjuvant radiation therapy post-surgery. There was no statistically significant difference in baseline characteristics between training and validation cohorts (**Table 1**).

### Independent risk factors and prognostic nomogram for OS

**Figure 2:**
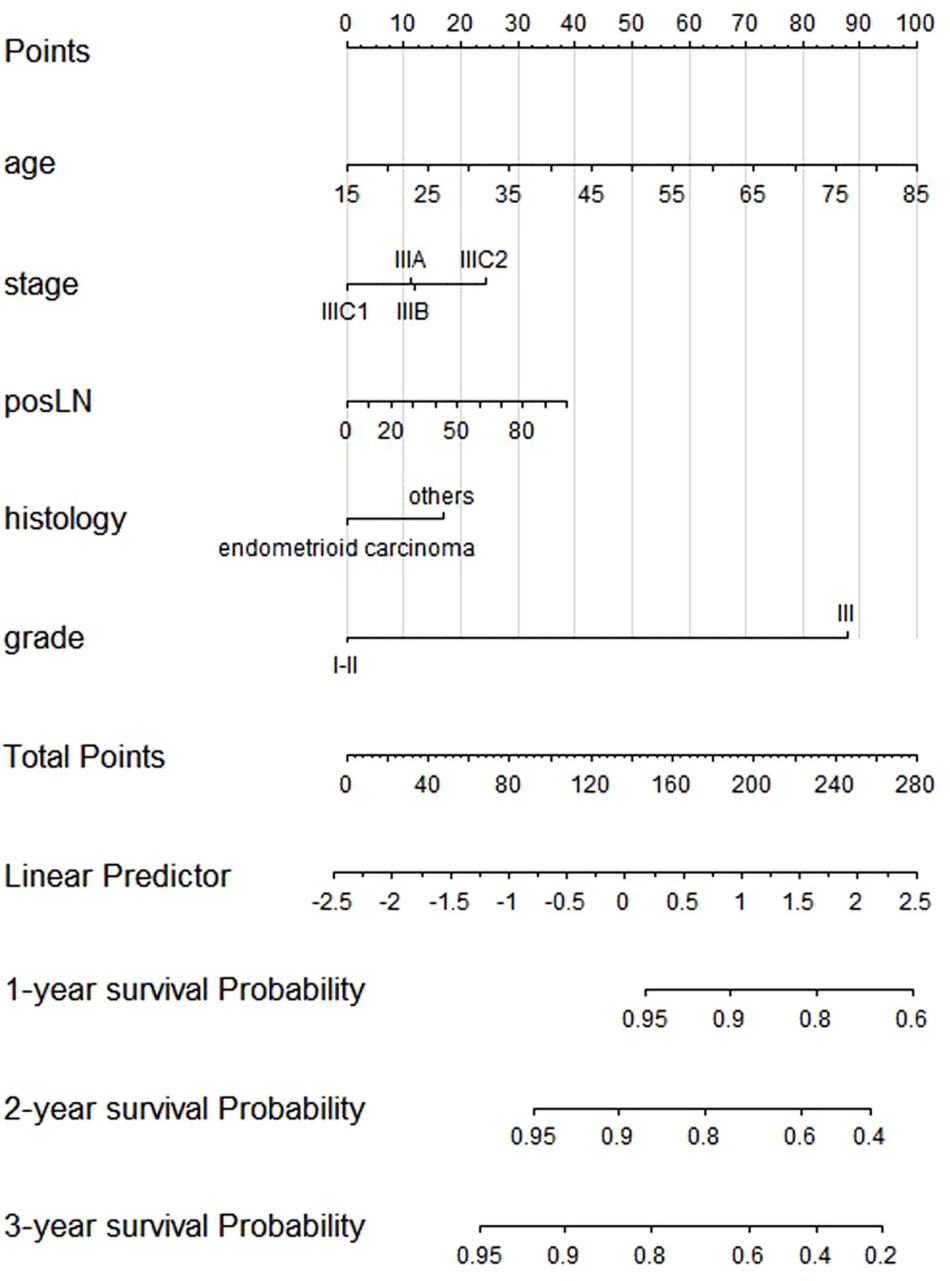
Nomogram of the composite model predicting 1-, 2-, and 3-year overall survival.

**Figure 3:**
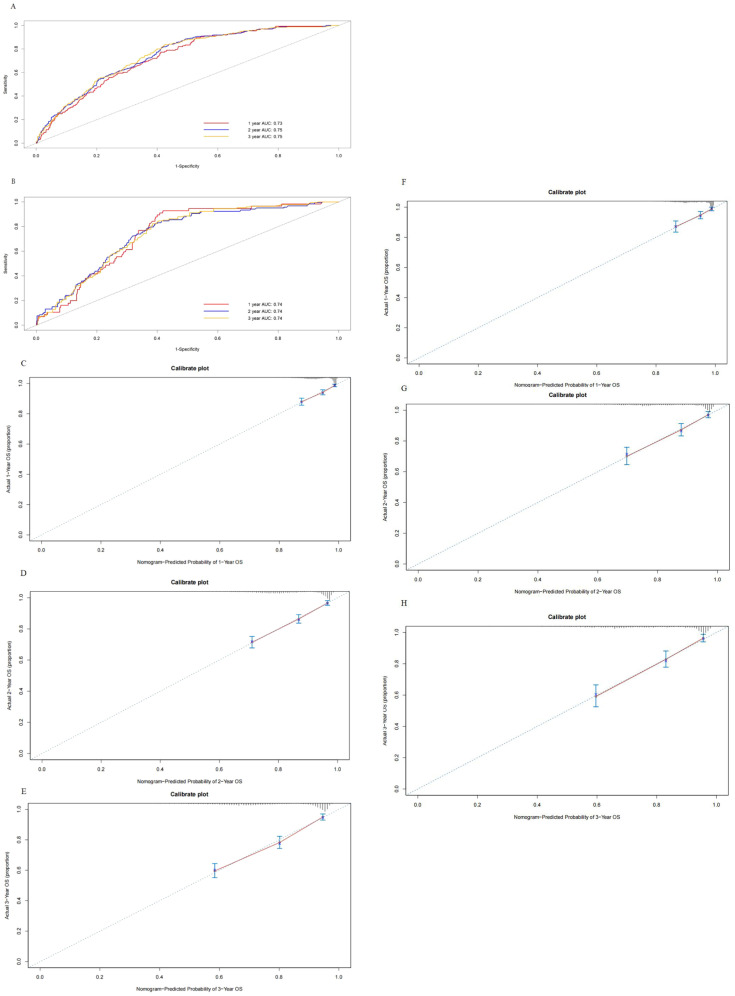
Receiver operating characteristics to describe the predictive power of the model in the training cohort **(A)** and the testing cohort **(B)**. Calibration curves of 1-year **(C)**, 2-year **(D)**, and 3-year overall survival **(E)** for stage III endometrial cancer patients in the training cohort. Calibration curves of 1-year **(F)**, 2-year **(G)**, and 3-year OS **(H)** for stage III endometrial cancer patients in the testing cohort.

After patient selection, univariate COX regression analysis was conducted, revealing that race, age, stage, histology, grade, tumor size, and the number of positive lymph nodes all had analysis results with *P* < 0.05. These variables were then further analyzed through stepwise multivariable regression; five variables, including age, stage, histology, grade, and number of positive lymph nodes, were ultimately selected with *p* < 0.05 to establish a nomogram associated with OS (**Table 2**, **Figure 2**). The C-index for OS in the training and testing cohorts were 0.73 (95% confidence interval [CI], 0.70–0.75) and 0.73 (95% CI, 0.69–0.77), respectively, showing better discrimination than the FIGO stage, whose C-index for OS was 0.58 (95% CI, 0.55–0.61) and 0.60 (95% CI, 0.55–0.65), respectively. Additionally, ROC curves at 1-, 2-, and 3- year OS were plotted for the training and testing cohorts (**Figures 3A, B**), with their respective AUC values being 0.73, 0.75, and 0.75 versus 0.74, 0.74, and 0.74. Calibration curves for 1-, 2-, and 3-year predictions also demonstrated consistency between the model-predicted and -observed survival probabilities (**Figures 3C–H**, respectively).

**Table 2:** Univariate and multivariate Cox regression analysis on variables for the prediction of overall survival in the training cohort (*n* = 2,844).

	Univariate analysis			Multivariate analysis		
Variable	HR	95% CI	*p*-value	HR	95% CI	*p*-value
Race						
White	Ref	Ref				
Black	2.14	1.68–2.73	< 0.001			
Others	1.18	0.855–1.64	< 0.001			
Age	1.05	1.03–1.06	< 0.001	1.025	1.013–1.037	< 0.001
Time_treatment	0.999	0.996–1	0.56			
Stage						
IIIA	Ref	Ref		Ref	Ref	
IIIB	1.23	0.831–1.83	0.298	1.011	0.681–1.502	0.954
IIIC1	0.819	0.628–1.07	0.141	0.822	0.619–1.092	0.177
IIIC2	1.69	1.27–2.27	< 0.001	1.262	0.934–1.705	0.03
Histology						
Endometriod cacinoma	Ref	Ref		Ref	Ref	
Others	3.05	2.44–3.83	< 0.001	1.348	0.742–1.049	< 0.001
Grade						
I–II	Ref	Ref		Ref	Ref	
III	6.35	4.67–8.62	< 0.001	4.636	3.302–6.508	< 0.001
Tumor size	1.01	1–1.01	< 0.001			
LN_Sur	0.983	0.872–1.11	0.784			
PosPLN	1.01	0.998–1.02	0.118			
PosLN	1.01	1–1.01	< 0.001	1.006	1.003–1.010	< 0.001

Five independent factors were applied for Cox regression analysis to construct the risk score for OS. The risk score formula based on the five independent factors was as follows: risk score = age × 0.025 + stage IIIB × 0.0116 + stage IIIC1 × −0.1956 + stage IIIC2 × 0.2323 + posLN × 0.0068 + histology others × 0.2983 + grade III × 1.5338.

### External validation

**Figure 4:**
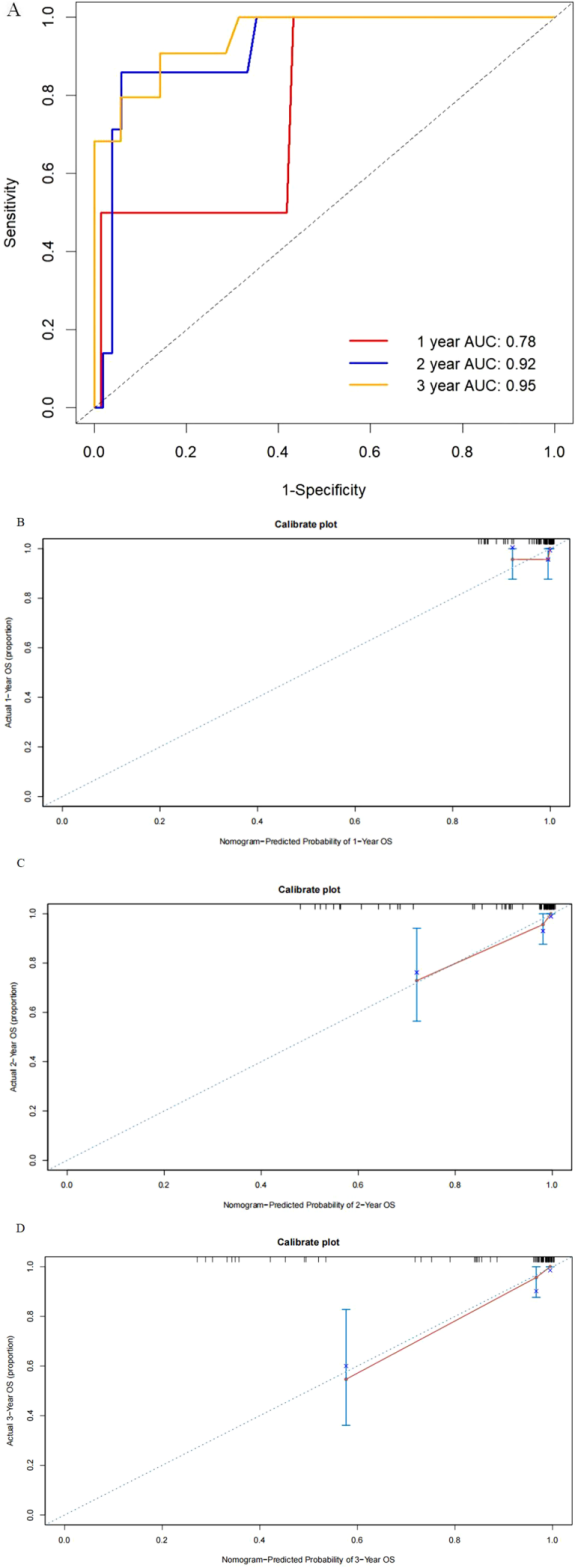
Receiver operating characteristics to describe the predictive power of the model **(A)** and calibration curves of 1-year **(B)**, 2-year **(C)**, and 3-year **(D)** overall survival for stage III endometrial cancer patients in the external validation cohort.

For the external validation cohort, the median follow-up time for these patients was 37 months (IQR, 24–59 months) longer than the training cohort, and the median age was 60 years (IQR, 53–65 years) similar with the training cohort. The distribution of pathological factors such as stage, grade, and histology was also similar to the SEER database. Next, five variables incorporating age, stage, histology, grade, and number of positive lymph nodes the same factors as the constructed nomogram in training were selected to establish the model. The model also showed exhibited discrimination for OS with a C-statistic value of 0.88 (95% CI, 0.78–0.97) and the predicted AUC was 0.78, 0.92, and 0.95 for 1-, 2-, and 3-year OS (**Figure 4A**), respectively. Calibration plots for the external validation demonstrated good consistency (**Figures 4B–D**).

### Interaction between radiotherapy and risk group

**Figure 5:**
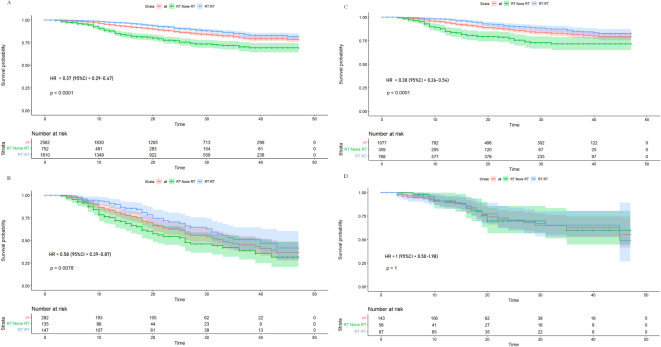
Kaplan–Meier overall survival curves for stage III endometrial cancer patients stratified by radiation in low-risk **(A)** and high-risk **(B)** patients from training cohort, and Kaplan–Meier overall survival curves for stage III endometrial cancer patients stratified by radiation in low-risk **(C)** and high-risk **(D)** patients from the testing cohort.

After the construction of the model and evaluation, the primary goal was the interaction between risk subgroups and radiation to identify individuals who benefit less from adjuvant radiotherapy among stage III EC patients. Therefore, in training and testing groups, we selected the optimal cutoff value for the risk scores predicted by the model and stratified patients into the low- and high-risk groups, with risk scores of ≤2.104863 and >2.104863, respectively. In training cohort, although the risk of death reduced significantly for the low-risk group (63% reduction, HR: 0.37, 95% CI: 0.29–0.47, *p* < 0.001) and the high-risk group (42% reduction, HR: 0.58, 95% CI: 0.39–0.87, *p* = 0.008), patients in the high-risk group had a less risk reduction from adjuvant radiotherapy compared with the low-risk group (p-interaction = 0.049) (**Figures 5A, B**). In the testing cohort, the risk of death reduced significantly in the low-risk group (62% reduction, HR: 0.38, 95% CI: 0.26–0.54, *p* < 0.001) but the benefit disappeared in the high-risk group (HR: 1, 95% CI: 0.50–1.98, *p* = 1), which showed the almost negligible risk reduction of adjuvant radiotherapy in the high-risk group (p-interaction = 0.016) (**Figures 5C, D**).

## Discussion

In this study, a predictive model for OS evaluated through external validation with five variables incorporating age, stage, grade, histology, and number of positive LN was conducted in stage III EC women to categorize individuals into the low- and high-risk groups, which was better than FIGO stage for prediction of OS. The selected high-risk group tended to benefit less from adjuvant radiotherapy than the low-risk group validating in training and testing cohorts; the risk group-by-radiation interactions were significant (p-interaction = 0.049 and 0.016, respectively).

The issue of whether patients can benefit for OS from adjuvant radiotherapy remains debated. Through interaction between radiotherapy and risk group, this study showed that the low-risk group could benefit significantly for OS from adjuvant radiotherapy while not in the high-risk group of stage III EC patients (p-interaction was 0.049 and 0.016 in training and testing cohorts, respectively). This result was consistent with PORTEC-3 in acquiring an absolute benefit in stage III EC (5). The study identified patients who benefit less or even benefit none mixed in stage III EC patients, which may be the reason for different outcomes in different clinical trials regarding the ambiguous benefit for OS in stage III patients. The risk classification from our model may serve as a reference for evaluating women with stage III EC on whether to choose adjuvant radiotherapy. While the study suggests that high-risk patients may benefit less and exempt from adjuvant radiotherapy, several potential treatment options exist for this group which include more intensive surveillance and advanced systemic therapies such as novel immunotherapy based on molecular subtyping. In recent years, with the rapid development of cancer immunotherapy, researchers have started exploring immune checkpoint inhibitors, such as PD-1/PD-L1 inhibitors, in the treatment of EC. EC patients, particularly those with mismatch repair deficiency (dMMR) or high microsatellite instability (MSI-H), are considered more likely to respond well to immunotherapy (18).

Variables were adopted by the Postoperative Radiation Therapy in Endometrial Cancer (PORTEC) one classification, the Gynecologic Oncology Group (GOG) 99 classification, and the Survival Effect of Para-Aortic Lymphadenectomy in EC (SEPAL) classification in the past few years when researchers seeking methods to address the limitations of existing classifications and guide decision making (19–24). Lymphovascular space invasion (LVSI) status was unavailable by SEER. However, compared with them, we added the number of positive LN, although the stage and number of LN contain redundant information regarding LN status. Not only the strong predictive ability of the number of LN (25) but also a recent study showed that adding the number of positive LN to FIGO stage as a covariate increased the ability of prognosis for III–IV EC than FIGO stage with a C-index of 0.781 (95% CI: 0.774–0.787) versus 0.776 (95% CI: 0.770–0.783) of FIGO stage (26).

FIGO stage, as the most widely used prognostic classification for EC, stratified patients based on the extent of tumor invasion, the degree of regional lymph node involvement, and the distant metastases (27). However, a better nomogram for the prediction of OS was constructed by recent studies (28, 29). Abu-Rustum et al. introduced an individualized five-variable nomogram for estimating the 3-year OS post-surgery more accurately (with a C-index 0.746) than the FIGO stage and passed external validation in 2012 as Polterauer et al. In addition, based on the SEER database of 64,023 EC patients, Koskas et al. also developed a nomogram to predict the 3-year OS of them with the C-index 0.811 for the total population with better discrimination than FIGO (30). Our results are consistent with the study above with the priority of concentrating on stage III EC previous studies never involved in. Of note, the 2023 FIGO staging system was modified, incorporating factors such as molecular subtypes, LVSI status, grade, and histology, additionally with a better reflection of the complexity of several types of EC and our understanding of their potential biological behaviors.

There were also limitations in this study. First, the SEER database lacks critical clinical information such as LVSI status and molecular subtyping (e.g., p53 status, mismatch repair deficiencies). These molecular markers are important for accurate prognostication, and their absence reduces the model’s robustness. Future studies should include these factors through multicenter collaborations and more comprehensive clinical data to optimize the model’s predictive power. Moreover, due to limited data in the SEER database, detailed adverse events and their long-time effects on patients’ quality of life (QoL) data were not included in our current analysis. We will integrate them in our subsequent study for a more holistic view of treatment outcomes. Second, the study’s generalizability is limited by the predominantly White patient population in the SEER database, with smaller proportions of Asian and African American patients. This limits the model’s applicability to diverse populations. Additionally, the external validation cohort was small (69 patients), weakening confidence in the model’s generalizability. Larger trials involving more ethnically and geographically diverse populations are needed. Last, the reliance on retrospective data weakens the conclusions. Prospective clinical trials are necessary to validate the model’s utility in real-world settings and confirm its potential to improve patient outcomes.

## Conclusion

In this study, a five-variable nomogram was constructed to predict the survival probability for stage III EC patients who received adjuvant chemotherapy with a C-index of 0.73, which shows better discrimination than FIGO stage (C-index = 0.58). In addition, internal and external validation of this nomogram in testing cohort and our institution was conducted, respectively. Women identified as high risk based on the nomogram benefit less or none from adjuvant radiotherapy compared to low-risk women through interaction analysis (p-interaction = 0.049 and 0.016 in training and testing cohorts). The predictive model may serve as a useful reference for better guidance of adjuvant radiotherapy in stage III EC patients.

## Data Availability

Publicly available datasets were analyzed in this study. This data can be found here: www.seer.cancer.gov.
