# Supplementary material for: Guiding adjuvant radiotherapy in stage III endometrial cancer: a prognostic model based on SEER
Source: Front Oncol. 2024 Nov 14;14:1480102. doi: 10.3389/fonc.2024.1480102 (PMC11602650; doi:10.3389/fonc.2024.1480102)
Supplement: Supplementary file 1 [file Table1.docx]

**Table S1. The selection of covariates by COX regression.**

| **baseline** | **univariate COX p<0.1** | **multivariable COX p<0.05** |
| --- | --- | --- |
| age | age | age |
| race | race | × |
| time from diagnosis to treatment | × |  |
| 2018 FIGO stage | 2018 FIGO stage | 2018 FIGO stage |
| grade | grade | grade |
| histology | histology | histology |
| tumor size | tumor size | × |
| number of harvest LN | × |  |
| number of positive LN | number of positive LN | number of positive LN |
| number of possitive pelvic LN | × |  |
| adjuvant radiation information | × |  |
| overall survival | × |  |
| survival status | × |  |
